# Multidimensional Analysis of Twin Sets During an Intensive Week-Long Meditation Retreat: A Pilot Study

**DOI:** 10.1007/s12671-025-02584-x

**Published:** 2025-05-07

**Authors:** Juan P. Zuniga-Hertz, Sierra Simpson, Ramamurthy Chitetti, Chang Francis Hsu, Han-Ping Huang, Alex Jinich-Diamant, Andrei V. Chernov, Julie A. Onton, Raphael Cuomo, Joe Dispenza, Dylan Davis, Leonardo Christov-Moore, Nicco Reggente, Wanjun Gu, Mitchell Kong, Jacqueline A. Bonds, Jacqueline Maree, Tatum S. Simonson, Andrew C. Ahn, Michelle A. Poirier, Tobias Moeller-Bertram, Hemal H. Patel

**Affiliations:** 1 Veterans Affairs San Diego Healthcare System, San Diego, CA 92161 USA; 2 Department of Anesthesiology, University of California, San Diego, Biomedical Sciences Building, Mail Code 0629, 9500 Gilman Drive, La Jolla, CA 92093 - 0629 USA; 3 Labfront (Kiipo Co.), Boston, MA 02067 USA; 4 Institute for Neural Computation, University of California, San Diego, La Jolla, CA 92093 USA; 5 Metamorphosis, LLC, Rainier, WA 98576 USA; 6 Department of Radiology and Biomedical Imaging, University of California, San Francisco, San Francisco, CA 94143 USA; 7 Institute for Advanced Consciousness Studies, Santa Monica, CA 90403 USA; 8 Division of Pulmonary, Critical Care and Sleep Medicine and Physiology, Department of Medicine, School of Medicine, University of California, San Diego, La Jolla, CA USA; 9 VitaMed Research, Palm Desert, CA 92260 USA

**Keywords:** Meditation, Mind–body practices, Quantitative electroencephalography (qEEG), Brain activity, Gene expression, Metabolomics

## Abstract

**Objectives:**

Meditation has long been known to promote health. We utilized a multidisciplinary approach to investigate the impact of mind–body interventions on the body in a twin cohort during a week-long meditation retreat.

**Method:**

This study was designed to address individual changes controlling for intersubject trait variation and explore the role of genetic background on multi-omic factors during meditation. Transcriptomic analysis was carried out from whole blood samples, while metabolomic and biochemical studies were carried out in blood plasma. Quantitative electroencephalography studies, coupled with biometric analysis and molecular studies at multiple time points, were carried out in twins meditating together and in twins separated and simultaneously either meditating or listening to a documentary.

**Results:**

Changes in gene expression, metabolites, and cytokines in blood plasma associated with specific meditative states showed patterns of change relative to the time point being assessed. Twin sets were similar in multiple domains before the start of the retreat, showed considerable divergence at the mid-point, and looked more similar by the end of the retreat. Twin pairs showed significant spectral power correlations in separate rooms and when only one twin meditated. These similarities were not observed in mismatched twin pairs. Heart rate dynamics assessments showed alignment among twin pairs, absent between unmatched pairs.

**Conclusions:**

To our knowledge, this pilot study is novel within the twin research paradigm and is a first step toward exploring the effects of meditation in twins.

**Preregistration:**

This study was not preregistered and was carried out under IRB protocol MED02#20211477.

**Supplementary Information:**

The online version contains supplementary material available at 10.1007/s12671-025-02584-x.

Chronic stress has been shown to have a profound negative effect on the body at psychological, physiological, and molecular levels. In particular, exposure to chronic psychosocial adversity can lead to marked changes in brain structure (Gianaros et al., 2007), gene expression (Cole, 2014), and epigenetic profile (Matosin et al., 2017). These changes can give rise to several diseases, including cancer, cardiovascular disease, and autoimmune disease (Cohen et al., 2007), as well as a host of psychological disorders, including anxiety, depression, post-traumatic stress disorder (PTSD) (Cohen et al., 2015), and addiction (Sinha & Jastreboff, 2013). At the molecular and cellular level, long-term stress can lead to a common molecular pattern known as “Conserved Transcriptional Response to Adversity” or CRTA (Cole, 2014). CRTA is the phenomenon used to describe the observation that genes upregulated in response to a diverse array of adverse social conditions, including chronic stress, were found to play a central pro-inflammatory role, while genes downregulated were involved in type 1 interferon innate antiviral responses as well as in IgG antibody synthesis (Cole, 2014).

Mind–body interventions (MBIs) such as meditation can improve the stress response in healthy and clinical populations (Wenuganen et al., 2021), leading to various health benefits. A recent study by Wenuganen et al. (2021) provides evidence for preventing the harmful effects of stress with a decades-long meditation practice. A randomized controlled trial carried out by Antoni et al. (2012) focused on cognitive-behavioral stress management (CBSM) demonstrated that even a short 10-week intervention was sufficient to reverse the upregulation of pro-inflammatory genes in an early-stage breast cancer cohort. Thus, it is tempting to speculate that meditation may create a new blood and tissue environment that can promote health resilience as supported by a recent study from our group showing meditators induced blood factors that prevented the entry of pseudotyped viruses for SARS-CoV-2 spike protein in cultured human lung cells (Zuniga-Hertz et al., 2023). Several additional studies have provided evidence for specific molecular changes associated with meditation and other MBIs (reviewed in Buric et al. (2017) and Muehsam et al. (2017)). Nevertheless, a multi-omic approach could potentially provide valuable insight into the molecular mechanisms by which meditation and other MBIs can improve health.

Twin studies represent a classic model for investigating genetic and environmental contributions to complex traits and phenotypes. To date, twin research has shown that brain activity, as well as many biological phenotypes, is strongly influenced by genetics (reviewed in van Dongen et al. (2012)). In the present study, we used a multidisciplinary approach to explore the effect of intensive meditation on molecular, physiological, and neurological correlates in a small twin cohort. Using a mixed sample of five sets of monozygotic (MZ) twin pairs and one dizygotic (DZ) twin set, we carried out quantitative EEG (qEEG) studies to monitor brain activity during an intensive 7-day meditation retreat. Heart rate (HR) data were continuously monitored throughout the week to determine HR dynamics in each twin set. Matched twin sets were compared with scrambled twin sets and age-matched non-twin controls. Finally, transcriptomics, metabolomics, and an exploratory panel of cytokines and other immune-related markers were used to assess meditation-specific molecular changes.

Pilot studies, conducted on a smaller scale than larger research studies, determine feasibility, guide decisions on whether to proceed, and clarify how best to conduct the larger study (In, 2017). They also evaluate safety, test recruitment strategies, refine randomization and blinding processes, and help calculate sample sizes (In, 2017). To our knowledge, this pilot study designed to determine feasibility and test aspects of recruitment, safety, randomization, and blinding represents the first of its kind aimed at addressing meditation effects in twins and provides the basis for a future study that includes a larger cohort of MZ and DZ twins sets.

## Method

### Participants

#### Recruitment

Formal invitation letters were sent to interested participants who learned of the study via a newsletter on the Inner Science Research Fund (ISRF) website. All twin participants were required to be at least 21 years of age and willing to attend the 7-day meditation retreat with their co-twin, provide blood samples, wear a Garmin wristwatch throughout the week, and agree to be brain-mapped for qEEG studies. Participants were not compensated for participating in the study. A total of six twin sets were recruited (Table 1) for a total of 12 participants. The age range was 25 to 76 (mean for twin age = 51.7 years, *SD* + / − 17.6 years). Each participant had varied meditation experiences. Data collected on a small group of healthy meditators participating in the retreat and part of a different study (manuscript in preparation) were used as non-twin controls.

**Table 1:** Twin pair demographics and data collection

Twin pair	Zygosity	Age*	Gender	Blood	Heart rate	qEEG
A	MZ	25	F	✓	+	+
B	MZ	63	F	+	+	-
C	MZ	56	M	✓	✓	✓
D	DZ	76	F	✓	✓	✓
E	MZ	36	F	✓	-	+
F	MZ	54	F	+	-	+

^*^Mean age = 51.7 years, *SD* + / − 17.6 years

✓, data available, complete

+ , data available, incomplete

-, data not available

### Procedure

The Advanced Week-Long Meditation Retreat was conducted in April 2022 at the Manchester Grand Hyatt Convention Center (San Diego, CA) and led by Joe Dispenza. This retreat has been previously described in detail (Zuniga-Hertz et al., 2023).

### Measures

#### Twin Study Design

**Fig. 1:**
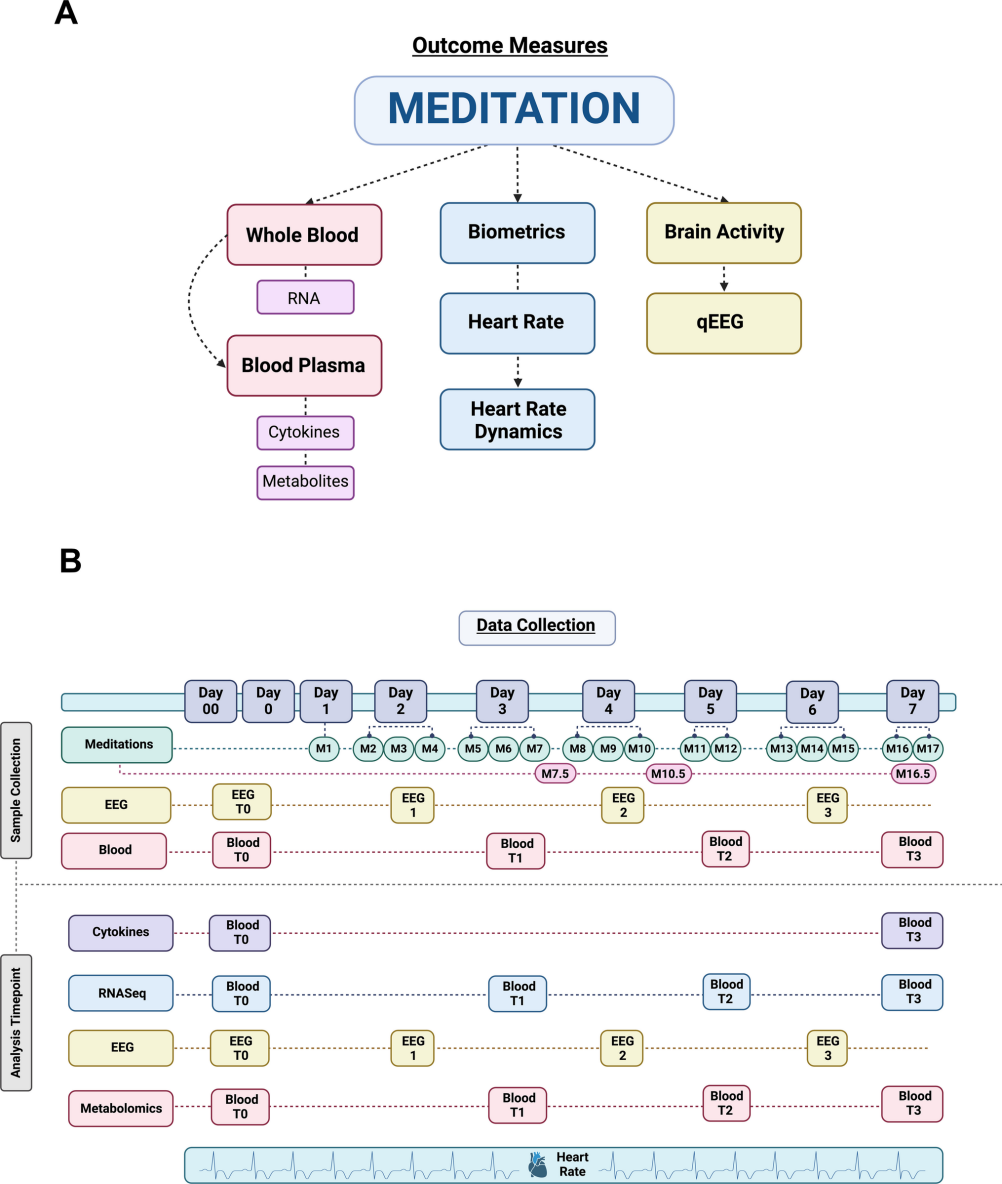
Schematic representation of twin study experimental design. **A** Outcome measures. **B** Timeline of meditation and data collection schedule for the 7-day meditation retreat. The retreat included 17 meditations (M1-17). Created with BioRender.com

To explore the effect of meditation in a twin research paradigm, we recruited six twin pairs. Outcome measures included analysis of RNA from whole blood, cytokines, and metabolites from blood plasma, biometric studies emphasizing heart rate dynamics, and brain activity as measured by qEEG (Fig. 1A). Data collection, summarized in Fig. 1B, began as early as 2 days before the retreat (Day 00). They included continuous heart rate monitoring using a Garmin wrist device, baseline blood collection, and qEEG recordings from each twin participant. Beginning on Day 2 of the retreat and including Days 4 and 6, one twin from each twin pair was separated from their co-twin during one retreat meditation and provided an audio recording of a documentary (as described below). Brain mapping was carried out for each meditating and non-meditating co-twin in addition to a 24-hr post-meditation blood sample to assess differences in blood environment within and among the twin pairs. On retreat Days 1, 3, 5, and 7, all twin participants were allowed to participate in all daily meditations.

#### Human Whole Blood and Plasma Collection

All blood samples were collected by a team of registered nurses, physicians, or phlebotomists 1–2 days before the retreat (baseline/T0; Fig. 1B) at an off-site location (2 days prior, Day 00; 2–4 pm local time) or on-site (1 day prior, Day 0; 11 am–4 pm local time). Three blood samples were collected on-site 24 hr after each retreat brain mapping session, including one on day 3 (T1 (Fig. 1B); 12:30 or 4:30 pm local time), a second on Day 5 (T2 (Fig. 1B); 12:30 or 4:30 pm local time), and a third on Day 7 (T3 (Fig. 1B); 2–5 pm local time). Study participants fasted for at least 30 min before providing a blood sample. Blood samples were obtained by venous puncture and collected in BD vacutainer K2 EDTA (for isolation of plasma) or BD PAXgene™ (for isolation of RNA) tubes (BD, Franklin Lakes, NJ). Blood samples were kept on wet ice for no longer than 30 min and frozen on dry ice (for RNA-seq studies) or centrifuged at 3000 RPM for 15 min (for metabolite and cytokine analyses) in an E8 Touch tabletop centrifuge (LW Scientific, Lawrenceville, GA) to isolate blood plasma. All blood and plasma samples were stored on dry ice until being shipped to UC San Diego at the end of the meditation retreat and stored at − 80 °C.

### Data Analyses

#### RNA-seq Studies

RNA-seq libraries were prepared with Illumina Stranded Total RNA Prep. Ribo-Zero Plus was applied to deplete abundant transcripts, including human cytoplasmic and mitochondria rRNA, and human beta-globin mRNAs. High-throughput sequencing was conducted at the IGM Genomics Center (UCSD) on the Illumina NovaSeq 6000. RNA-seq data (25–30 million reads/sample) were saved in FASTQ format. Due to the poor quality of the sample from twin group C (male MZ twin pair), these data were excluded from analyses.

#### Transcriptome Analysis

Files saved in FASTQ format were pre-filtered to remove low-quality bases, TruSeq dual-index adapter sequences, and unpaired reads using *Trimmomatic* (Bolger et al., 2014). Transcript-level mapping and gene-level quantification were conducted in STAR aligner using human reference genome and transcriptome databases (Gencode 43 (GRCh38.p13)). The quality of RNA-seq, mapping, and quantification was assessed using *FastQC* (Babraham Bioinformatics) and *MultiQC* (Ewels et al., 2016) tools. Samples with quality scores identified below thresholds were excluded.

Gene-level quantification and annotation were done using *Tximeta* utility (Love et al., 2020). Gene count matrices were normalized, and differential expression analyses were done in *DESeq2* (Love et al., 2014). Outlier samples were detected and excluded using Cook’s distance method. The adjusted log_2_FC values were calculated using the adaptive *t*-prior *apeglm* method (Zhu et al., 2019). Significant differentially expressed genes (DEGs) were defined by Wald’s test *p* < 0.05 and *p*_adj_ < 0.1 (or both) and used in downstream analyses. Batch effects were controlled using *removeBatchEffect* (Ritchie et al., 2015) and *RUVseq* (Risso et al., 2014) functions. DEGs were visualized using principal component analysis (PCA) tools, *ComplexHeatmap*, and *EnhancedVolcano* Bioconductor packages. DEGs were determined using DESeq2 and a generalized linear model in which counts for each gene and sample were modeled using a negative binomial distribution with fitted means and gene-specific dispersions (Love et al., 2014). Fold changes (FC) were calculated by Wald’s test with respective *p*-values (*p* < 0.05). Thresholds of *p* < 0.05 and FC > 1.5 (upregulated DEGs) or FC < − 1.5 (downregulated DEGs) (or log_2_FC > 0.58 and log_2_FC < − 0.58, respectively) were applied to increase the specificity of downstream analyses. Bioinformatic software and resources are summarized in Supplementary Table S1.

Biological interpretations of signaling pathways and Gene Ontology (GO) terms were conducted using default parameters in clusterProfiler (Wu et al., 2021) based on significance scores (*p*-values < 0.05) calculated by the Fisher exact test (Wu et al., 2021). Kyoto Encyclopedia of Genes and Genomes (KEGG) (Kanehisa et al., 2023) and GO public databases (Ashburner et al., 2000) were used for data mining. To increase the accuracy of FC estimates for genes with low counts and high dispersion values, log_2_FC estimates were shrunken toward the prior using the *apeglm* algorithm (Zhu et al., 2019).

#### Cytokines, Biologically Active Enzymes, and Growth Factor Exploratory Panel

To profile cytokines, biologically active enzymes, and growth factors in twin participant plasma samples, we used a Proteome Profiler^TM^Assay kit (R&D Systems, Minneapolis, MN). Briefly, the membranes were incubated with 200 µL plasma diluted in Array Buffer 6 to a final volume of 1500 µL and incubated overnight at 4 °C with continuous mixing. Samples corresponding to baseline (pre-retreat) and T3 (post-retreat) time points were analyzed for each twin participant. Membranes were processed following the manufacturer’s protocol and cytokine levels were quantified in a UVP unit (UVP BioLite™ MultiSpectral Source). Data is presented as a delta of the percentage of change before and after the retreat (T3 — baseline/T0). All samples were carried out in duplicate and averaged before the delta calculation.

#### Human Plasma Metabolomics

Global metabolomic profiling of plasma was performed using the non-targeted Metabolon platform (Metabolon Inc., Morrisville, NC). Frozen plasma samples were extracted with 95% methanol to precipitate proteins and remove small molecules, followed by centrifugation (20,000 rpm/15 min/4 °C) to precipitate larger proteins. The resulting supernatants were analyzed by Waters ACQUITY ultra-high performance liquid chromatography-tandem mass spectroscopy (UPLC-MS) and a Thermo Scientific Q-Exactive high-resolution mass spectrometer. Metabolites were identified by comparing the ion features to a reference library of standards, including molecular weight (M/Z), retention time, and the MS/MS data.

#### Heart Rate Dynamics Studies

All study participants were given a Garmin VivoSmart 4 (Garmin, Olathe, Kansas) wristwatch before the start of the retreat. As part of the set-up process, each participant was required to download Garmin Connect and PhysioQ apps onto their iPhone or Android devices. Beat-to-beat interval (BBI) data were collected continuously throughout the 7-day retreat by photoplethysmography. Labfront Analytics, LLC provided all data storage and analysis.

A total of 357 BBI segments were analyzed (17 meditation periods × 21 participants = 357 recordings). The 21 participants included three MZ twin pairs, one DZ twin pair, and 13 age-matched controls who underwent the same meditation protocols. Data from the two other MZ twin pairs were missing and unavailable for analysis. The normality of age distributions was assessed using the Kolmogorov–Smirnov test, and differences in ages between groups were compared with *t*-tests. The BBI processing method consisted of three steps. First, data were preprocessed to detect and correct outliers in the BBI series (Lipponen & Tarvainen, 2019). Next, the piecewise cubic Hermite interpolating polynomial method was applied to transform the BBI tachogram into a BBI time series with a sampling frequency of 1 Hz. Lastly, a 10-s moving window was applied to smooth the BBI series.

In a Pearson correlation analysis, two participants’ BBI time series from the same meditation period were aligned in time and compared using a 300-s sliding window with a 60-s hop length. Pearson correlation coefficients were calculated for each hop between the BBI segments within the window. These coefficients were then averaged to represent the overall Pearson correlation between the two BBI series in the same meditation period. Similarly, the absolute difference in BBI was calculated across each data point within the meditation period and then averaged.

Pairwise comparisons were calculated using four different combinations: between monozygotic twin pairs (T), between dizygotic pairs (DT), between twins from different sets of pairs (N) — e.g., a twin from MZ-pair B and a twin from MZ-pair A, and between a twin and an age-matched control (C). To carry out these comparisons, the BBI data from each of the 17 meditations were evaluated separately to determine the HR dynamic concordance (Pearson correlation and BBI absolute difference) from the paired individuals. In some of these meditation periods, one of the twins was seated in a different room, separated from their co-twin, and thus removed from any ongoing meditation activities. For this scenario and for times when data were missing or filled with noisy artifacts, the data were excluded from any potential pair-wise comparisons (summarized in Supplementary Table S2). The total number of meditation data pairings in the twin group included in the analyses totaled 276, while the total number of data pairings in the control group totaled 246.

#### Quantitative Electroencephalography

Each twin set had a baseline recording on Day 00–Day 1 of the 7-day retreat. The baseline recording consisted of 5 min of resting state eyes open, 5 min of resting state eyes closed, and a 15-min meditation. This recording served as the baseline for evaluation of each brain mapping meditation that occurred during the retreat. For brain mapping meditations, twin sets were split for three meditations each, whereby one twin was meditating while their co-twin was in a separate room listening to an audio documentary on the Mariana Trench (Hirose, 2017).

For each brain mapping session, EEG data was recorded with the mBrain Train Smarting headset, comprised of a 32-channel EEG system positioned according to the international 10/20 system. The impedance per electrode was maintained under 20k ohms, and a 0.5% saline solution was used to reduce electrode–skin impedance. The signal was recorded at a 2000-kHz sampling rate with a reference at FCz.

EEG data from the five MZ twin pairs were analyzed to simplify data interpretation. Data were imported into Matlab (Mathworks, Natick, MA, USA), a high-pass filter was applied above 0.5 Hz, 60 Hz artifact was removed using cleanline (EEGLAB, UCSD), and bad channels, resulting from excess noise and leading to loss of signal, were interpolated. Data were epoched into small windows for artifact rejection via a probability algorithm in EEGLAB, and independent component analysis (ICA) was run on the final EEG data. Independent components (ICs) were classified using ICLabel (EEGLAB, UCSD), which returns a probability of being a brain-derived signal or five different artifact categories. ICA weights were applied back to the continuous data, and ICs with over 80% probability of being eye or muscle artifacts were removed from the data.

Of the five twin pairs, only three had pre-baseline sessions in common, and there were eight meditation/documentary sessions across the five twin pairs. For each twin pair (and sham pair not related to each other), data lengths were equalized by cutting down the larger data set from the end, and data were then windowed into 1.5-s epochs using a sliding window every 0.1 s. Both data sets were subjected to automated probability calculations to determine noisy epochs. All marked epoch numbers were collected, pooled, and removed from each data set to maintain the same time points across participants. Each epoch was decomposed into frequency power between 1.5 and 50 Hz using three-cycle wavelets at the lowest frequency, 30 cycles at the highest frequency, and evenly distributed cycles in between. Power was averaged in six frequency bands: 1.5–4 Hz, 4–7 Hz, 7–12 Hz, 12–18 Hz, 18–30 Hz, and 30–50 Hz. For each channel, power in each band was smoothed over 2 min, and about 20 points were extracted from the resulting time course to show coarse-grained changes in power over time. Power at these time points for each band and channel was submitted to correlation analysis comparing the time course of each MZ twin (and sham) pair.

#### Data Analyses

For the cytokines, biologically active enzymes, and growth factor exploratory panel, Welch’s two-sample *t*-test was used. To assess effect size, Hedges’ *g* was utilized. To verify equal variances, Levene’s test was carried out. For metabolomics studies, features were batch normalized, log-transformed (base 10), and auto-scaled (mean-centered and divided by the standard deviation of each variable); zeros or missing values were added, and standard statistical analyses were performed. Welch’s two-sample *t*-test, PCA, partial least squares-discriminant analysis (PLS-DA), and a one-way analysis of variance (ANOVA) were used for parametric data with Tukey’s post hoc, and Kruskal Wallis was used for non-parametric data. PLS-DA is performed using *Metaboanalyst* (Xia et al., 2009), a Java-based GUI for R and Bioconductor packages (Gentleman et al., 2004). Classification and tenfold cross-validation are performed using the corresponding wrapper in the *caret package* (Bijlsma et al., 2006). *p*-values were adjusted for multiple comparisons using the Benjamini–Hochberg false discovery rate (FDR). Correlation heatmaps were generated using Pearson *r* distance measures.

For heart rate dynamics studies, the degree of difference for BBI similarity indices between any two groups listed in Supplementary Table S2 was performed in terms of *p*-value using the Wilcoxon rank-sum test. In addition, to account for baseline variations, a mixed effects model was fit to assess differences in BBI absolute difference between groups with additional covariates for time and time by-group interactions. The statistical significance was set at *p* < 0.05. All data analyses were performed using Python’s open-source statistical package, SciPy v1.10.1 (Virtanen et al., 2020).

For qEEG studies, correlation *R*-values from twin pair power time courses were averaged for all twin pairs for the baseline/T0 and meditation/documentary conditions. To calculate significance, sham pairings were created for all possible non-twin pairs in baseline/T0 and meditation/documentary conditions, resulting in 12 sham pairs for each condition. For the baseline/T0 condition, bootstrap *R*-value means for each channel and frequency band were derived from 1000 random selections of three non-twin pair correlations. For the meditation/documentary condition, bootstrap *R*-value means were derived from 1000 random selections of eight non-twin pair correlations. Each resulting collection of mean *R*-values was sorted, and the values at 5% probability from either end of the distributions were selected as the masking limits for the real twin pair means (*p* = 0.05). *R*-values for the real twin matrices between these minimum and maximum masking values were set to zero to indicate that they were not significantly different from correlations between unrelated pairs.

## Results

### Meditation-induced Changes in the Blood Environment in Twin Pairs During a 7-day Intensive Meditation Retreat

To explore the effect of meditation in a twin research paradigm, we carried out an exploratory multi-omic analysis of blood in a twin cohort (Fig. 1A). Outcome measures included RNA analysis of whole blood and a panel of cytokines, biologically active enzymes, and growth factors from blood plasma. Finally, we performed metabolomic analysis of twin blood plasma.

### RNA-seq and Differential Gene Expression Analysis

**Fig. 2:**
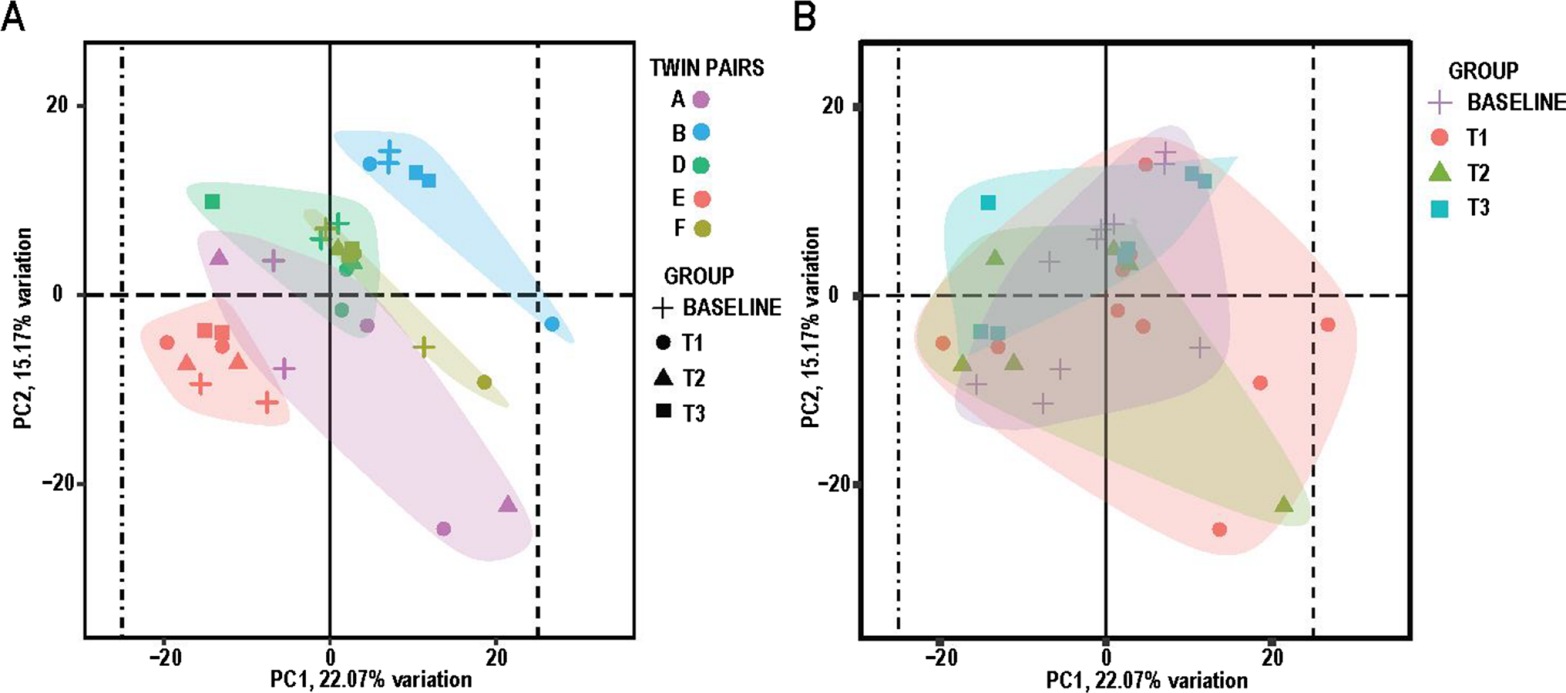
Principal component analysis (PCA) of normalized genes. **A** Clustering by twin pair IDs. **B** Clustering by baseline, T1, T2, and T3 groups. Colored areas indicate cluster overlaps according to respective legends. Baseline, *n* = 10; T1, *n* = 10; T2, *n* = 6; T3, *n* = 7

Transcriptome analysis (RNA-seq) was carried out using total RNAs isolated from blood samples of six pairs of twins. Following RNA quality assessment, twin pair C (male MZ twin pair) was excluded from the analysis. To enable comparative transcriptome analysis, the dependence of the variance on the mean and library size was removed by gene count normalization using the variance stabilizing transformation (VST) (Anders & Huber, 2010; Tibshirani, 1988) integrated into the standard DESeq2 bioinformatic workflow (Love et al., 2014). PCA applied to the VST-converted count matrix revealed that individual twin pairs exhibited similar transcriptional profiles while variability was observed among twin pairs (Fig. 2A). A difference was observed in time-dependent groups T1, T2, and T3 relative to baseline/T0 as demonstrated by the partial overlap of respective.

**Fig. 3:**
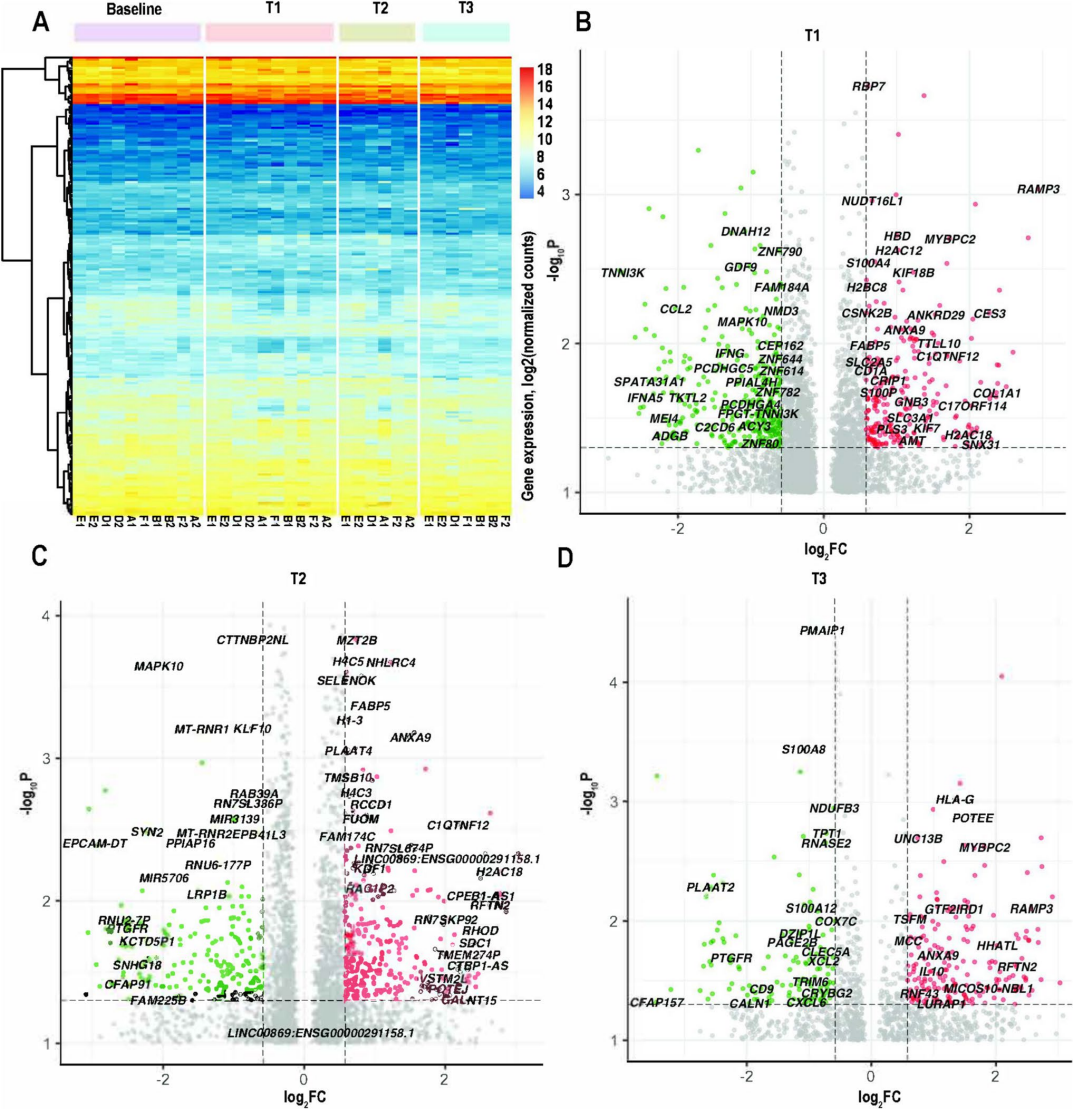
Transcriptomic changes as a function of time. **A** Hierarchical clustering analysis of the differentially expressed genes ranked by decreasing variance across groups and biological replicates (FC > 1.5, *p*_adj_ < 0.1, baseline *n* = 10 samples/group; T1 *n* = 10 samples/group; T2 *n* = 6 samples/group; T3 *n* = 7 samples/group). Heatmap colors correspond to absolute gene expression (log_2_ (normalized counts)): blue, yellow, and red correspond to low to high gene expression according to the scale. **B**–**D** Volcano scatter plots (− log_10_*p* vs. log_2_FC) of significant DEGs in T1 (**B**), T2 (**C**), and T3 (**D**) relative to baseline/T0. Red and green colors indicate up- and downregulated DEGs, respectively. Gray dots — genes below significance and FC thresholds. Thresholds (log_2_FC > 0.58 or log_2_FC < − 0.58) and − log_10_*p* < 0.05 are indicated by dashed lines. Selected DEGs are labeled

PC1/PC2 projections (Fig. 2B). These data demonstrate a large number of gene changes at the beginning of the retreat which normalized by the end of the retreat. Hierarchical clustering analysis of the top genes ranked by decreasing variance presented clusters of co-expressed genes across time-dependent groups and biological replicates (Fig. 3A). Significant up- and downregulated protein-coding DEGs are presented on volcano scatter plots for all time-dependent comparison groups (Fig. 3B–D, Supplementary Table S3).

### Gene Ontology Analysis

**Fig. 4:**
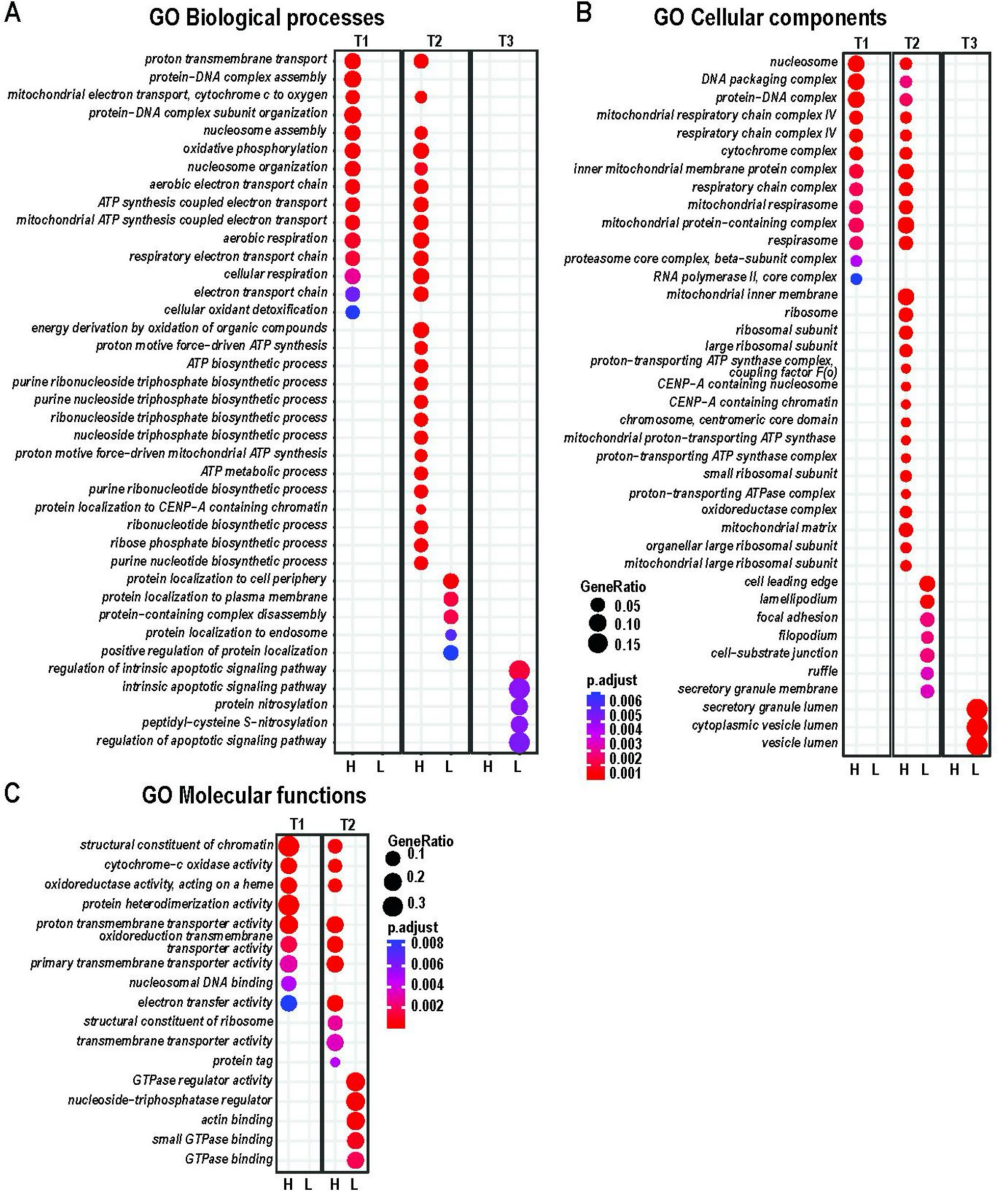
Cluster analysis by Gene Ontology (GO). Biological processes (**A**), cellular components (**B**), and molecular functions (**C**) were identified by enrichment analysis of significant DEGs (*p* < 0.05) in T1, T2, and T3 relative to baseline/T0. Circle size corresponds to gene ratios (a fraction of differentially expressed genes found in the gene set) and the Fisher exact test *p* (*p* < 0.05, blue to red colors — lower to higher significance) according to the scales. H/L columns: GO term is predicted to have higher (H) or lower (L) activity relative to baseline/T0. Lists of GO terms and statistical parameters are included in Supplementary Table S3

Enrichment analyses of protein-coding and non-coding RNA (ncRNAs) DEGs annotated in the GO database and fitting the standard statistical criteria (*p* < 0.05, |log_2_FC|> 0.58) were conducted to estimate significant GO biological processes, cellular components, and molecular functions in each T1 vs. baseline/T0, T2 vs. baseline/T0, and T3 vs. baseline/T0 comparison groups (Fig. 4A–C, Supplementary Table S4). Studies of GO enrichment predicted an increase in multiple mitochondria-specific activities, oxidative phosphorylation, ATP synthesis, and respiration processes in T1 and T2 groups (Fig. 4A, B). In T2, the elevation of ATP and ribonucleoside biosynthesis was detected. In addition, regulatory epigenetic biological processes and molecular functions, including nucleosome assembly and structural chromatin reorganization, were more active in T1 and T2 relative to baseline/T0. In T3, intrinsic apoptotic protein nitrosylation, secretory granules, and cytoplasmic vesicles demonstrated lower activity than baseline/T0. Taken together, these data indicate that the intensive retreat experience results in large changes in mitochondrial function, suggesting a shift in fuel sources.

### Blood Plasma Cytokines, Biologically Active Enzymes and Growth Factor Exploratory Panel

**Fig. 5:**
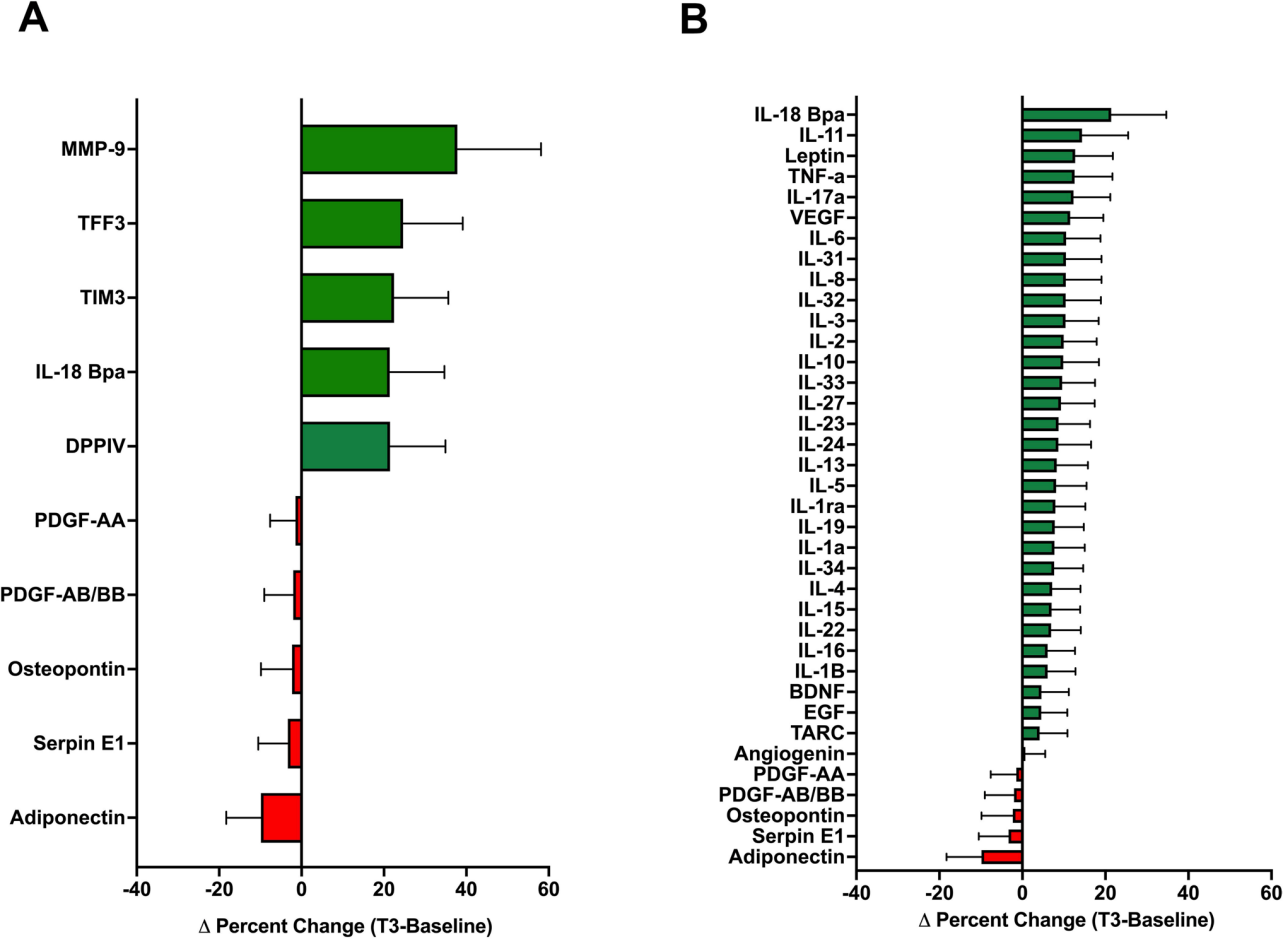
Changes in selected plasma cytokines, biologically active enzymes, and growth factors. **A** Percent change (T3 — baseline/T0) for top five upregulated (green) and downregulated (red) proteins. **B** Percent change (T3 — baseline/T0) for gut-brain axis-related cytokines and proteins showing upregulated (green) and downregulated (red) factors. Protein arrays were performed on blood collected at baseline/T0 (Days 00 or 0) and at T3 (Day 7)

Data were collected at two time points, pre-retreat (baseline/T0) and post-retreat (T3), and the delta (T3-baseline/T0) was determined for 105 cytokines, biologically active enzymes, and growth factors. Participant variability was observed among individuals and twin sets. Of all the biologically active proteins tested, only retinol-binding protein 4 (RBP-4) was significantly increased (136%, *t* (11) = 2.939, *p* = 0.036, Hedges’ *g* = 1.34) following the meditation intervention. The top five proteins with increasing trends included matrix metalloproteinase-9 (MMP-9; 245%), trefoil factor 3 (TFF3; 160%), T cell immunoglobulin and mucin domain 3 (TIM3; 146%), interleukin (IL)−18 binding protein (IL-18Bp; 139%), and dipeptidyl peptidase IV (DPPIV; 139%). Trending decreases were observed in only five of the 105 proteins tested (Fig. 5A) and included platelet-derived growth factor-AA (PDGF-AA; − 9%), platelet-derived growth factor-B (PGDF-BB; − 13%), osteopontin (OPN) (− 14%), serine protease inhibitor E1(SERPINE1) (− 21%), and adiponectin (− 64%). Trends were observed with overall increases in both pro- and anti-inflammatory interleukins (Fig. 5B). A comprehensive listing of changes showing both function and inflammatory potential is included in Supplementary Table S5. Taken together, these data suggest increased expression of proteins involved in regeneration and repair.

### Blood Plasma Metabolites

There have been few studies exploring the effect of meditation on circulating metabolites (Schoenberg & Gonzalez, 2022; Xue et al., 2018, 2022). Meditation practices also vary by length and difficulty, making a direct comparison challenging. A snapshot of biologically active metabolites was collected to better understand the effect of meditation on the periphery. Trends were explored in twin pairs and across time points. After sample processing and filtering, 896 metabolites were included in the analysis. To obtain a comprehensive understanding of the data set by twin pair and by time point, PLS-DA models were generated and the metabolites with variable importance in projection (VIP) scores > 2 were extracted as they are considered relevant to the model. An exhaustive list of PLS-DA scores can be found in Supplementary Table S6 (by time) and Supplementary Table S7 (by twin pair) as VIP > 1 is often considered important to the model. 

**Fig. 6:**
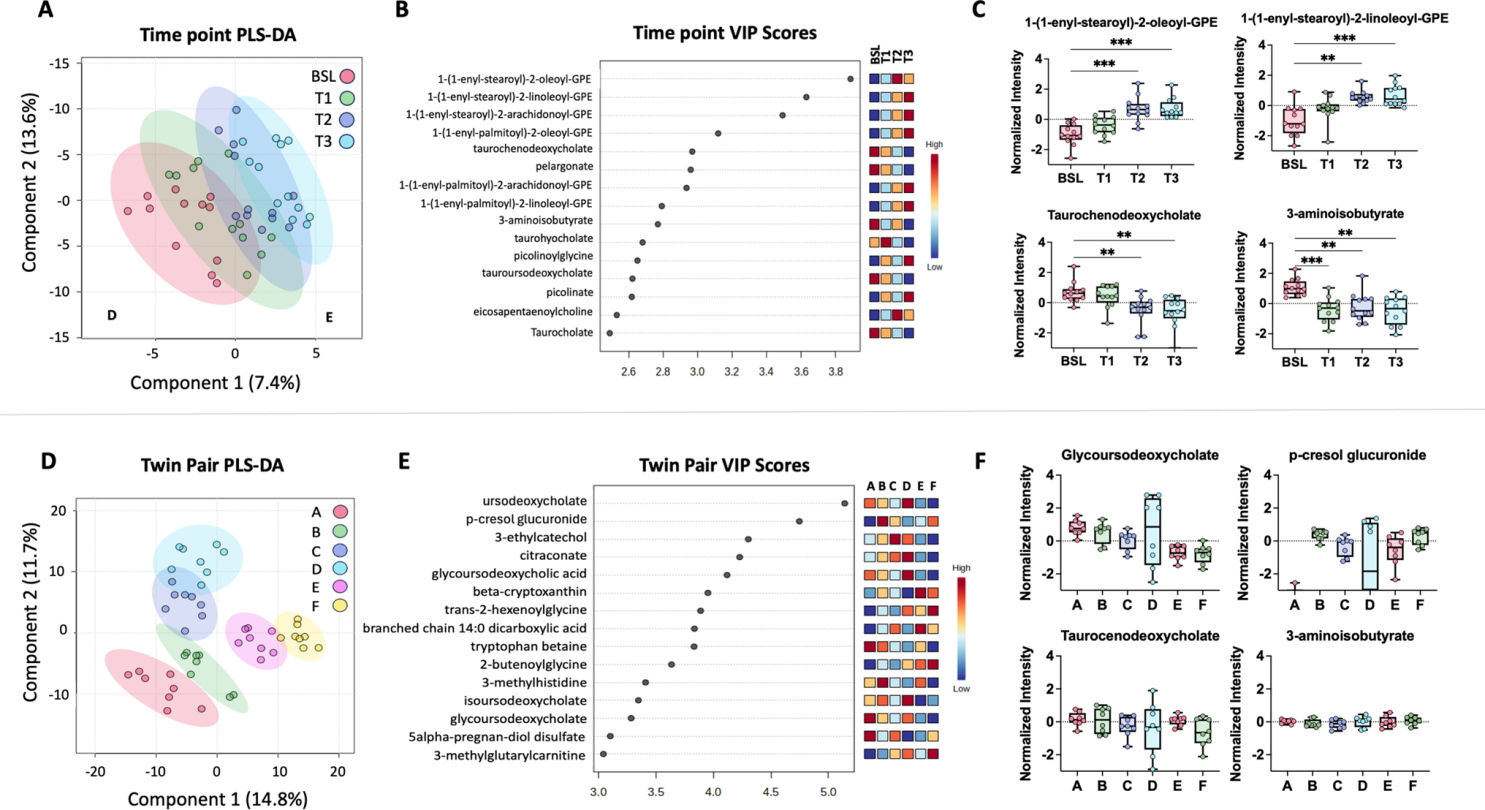
Top 15 significantly changed circulating plasma metabolites.** A** Time point partial least squares-discriminant analysis (PLS-DA) of metabolite changes for baseline (BSL)/T0 (pink), T1 (green), T2 (dark blue), and T3 (light blue). While some metabolites are present in baseline/T0 and T3 time points, significant overlap can be seen throughout the subsequent time points analyzed. **B** Time point variable importance in projection (VIP) scores indicate the top 15 metabolites changed throughout the retreat. **C** Box plots demonstrating statistically significant increases in 1-(1-enyl-stearoyl)−2-oleoyl-glycerophosphoethanolamine (GPE) and 1-(1-enyl-stearoyl)−2-linoleoyl-GPE and decreases in taurochenodeoxycholate/TCDCA and 3-aminoisobutyrate/BAIBA. **D** Twin pair PLS-DA analysis of metabolite changes in twin pairs A (pink), B (green), C (purple), D (light blue), E (magenta), and F (yellow). A strong clustering of metabolites by twin pair was observed. **E** Twin pair VIP scores for the top 15 metabolites changed during the retreat. **F** Box plots demonstrating statistically significant changes in glycoursodeoxycholate, p-cresol glucuronide, taurochenodeoxycholate/TCDCA, and 3-aminoisobutyrate/BAIBA

**Fig. 7:**
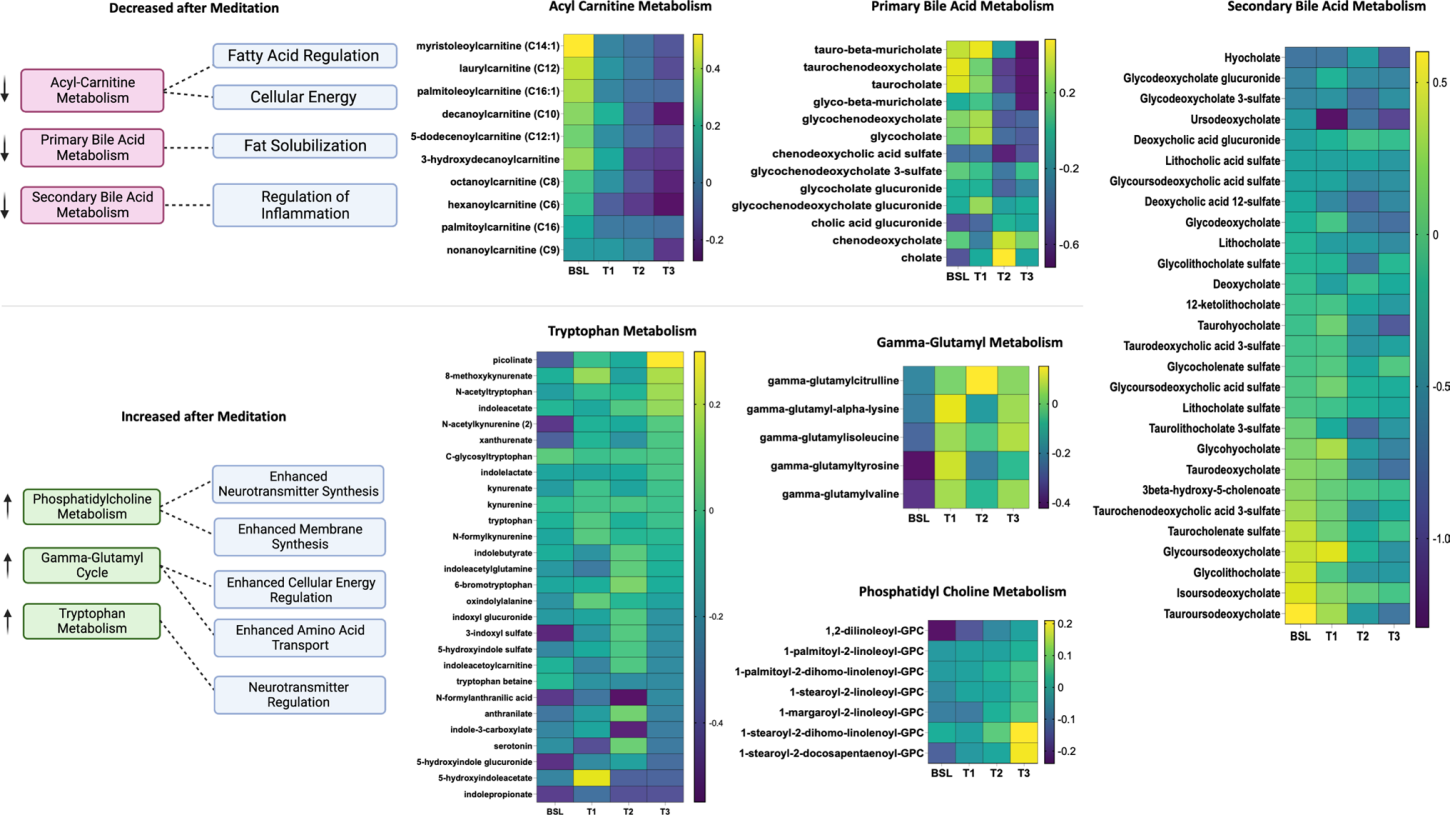
Heatmaps of meditation-induced plasma metabolite change over time. Metabolites are classed by relevant pathways, including fatty acid metabolism, primary bile acid metabolism, secondary bile acid metabolism, phosphatidylcholine metabolism, gamma-glutamyl cycle, and tryptophan metabolism. Pathways with an observed increase (pink) or decrease (green) are shown along with corresponding biological processes. BSL, baseline

Figure 6 shows metabolomic data from the meditating twin cohort. A graded change was observed over time (Fig. 6A), with the largest differences occurring at baseline/T0 and T3. The top 15 VIP scores revealed that multiple plasmalogens, including 1-(1-enyl-stearoyl)−2-oleoyl-glycerophosphoethanolamine (GPE) (Fig. 6B, C), 1-(1-enyl-stearoyl)−2-linoleoyl-GPE (Fig. 6B, C), and (1-(1-enyl-stearoyl)−2-arachidonyl-GPE) (Fig. 6B), were significantly increased (all *p* < 0.001) from baseline/T0 to T3. Plasmalogens are a subclass of cell membrane glycerophospholipids believed to play an important role in protecting cell membrane components from oxidative stress (Almsherqi, 2021). Significant decreases were observed in the bile acid taurocenodeoxycholate/taurochenodeoxycholic acid (TCDCA) (*p* = 0.003) (Fig. 6B, C). Additionally, 3-aminoisobutyrate (BAIBA), a beta amino acid involved in the breakdown of thymine, was also significantly decreased (*p* = 0.002) (Fig. 6B, C). Further exploration of the change from baseline/T0 to T3 revealed decreasing trends in acyl-carnitine metabolism, primary bile acid metabolism, secondary acid metabolism, and increases in gamma-glutamyl cycle, phosphatidylcholine (PC) metabolism, and tryptophan metabolism. Heat maps generated to visualize the trends of constituents of these sub-pathways are shown in Fig. 7. These data suggest that this form of meditative practice results in increased neurotransmitter and cellular membrane synthesis and changes in the regulation of these processes.

Twin pairs were combined across time and PLS-DA was used to classify each twin pair (Fig. 6D). VIP scores > 2 were used to identify features of interest. Overall, twin pairs clustered together despite all time points being included for each pair (Fig. 6D). Trends of differences were observed across twin pairs. The top VIP score was observed for ursodeoxycholate, a hydrophilic bile salt. This metabolite was significantly more abundant in twin pair A than in pair E (*p* = 0.006) and pair F (*p* < 0.001). Ursodeoxycholate was also more abundant in twin pair E than in pair F (*p* = 0.046). Next on this list is p-cresol glucuronide, a metabolite of p-cresol, which was significantly more abundant in twin pair A compared to B, C, E, and F (*p* < 0.001). While TCDCA and BAIBA were both significantly changed over time (Fig. 6B, D), these metabolites were not significantly changed between twin pairs (Fig. 6F). These data suggest that observed differences in TCDCA and BAIBA were not driven by twin pair differences but resulted from the intervention.

### Physiological Correlates in Twin Pairs During a 7-Day Intensive Meditation Retreat

**Fig. 8:**
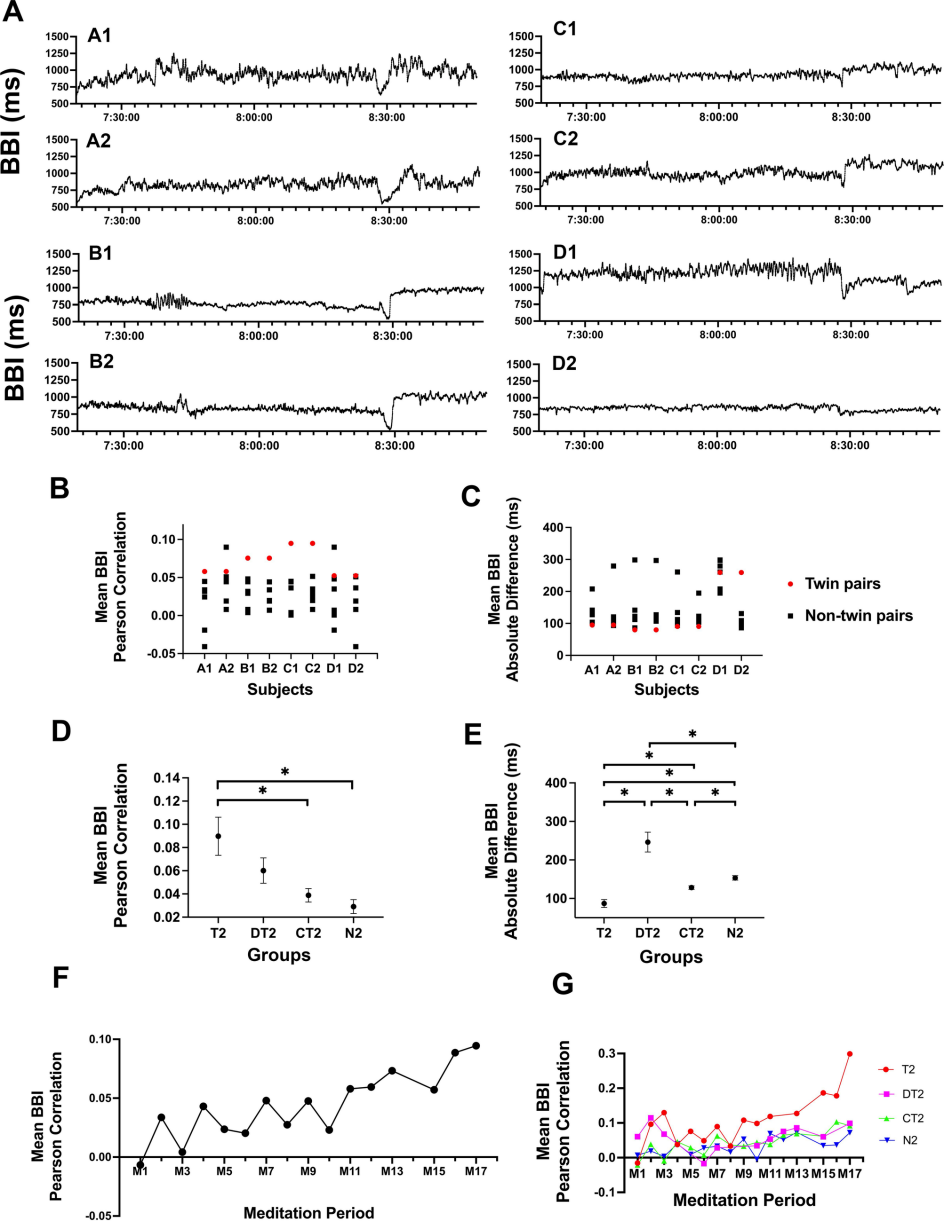
Heart rate dynamics in meditating monozygotic and dizygotic twins. **A** Comparison of BBI time series in four meditating twin pairs. (A), (B), and (C) correspond to monozygotic twin pairs, while (D) corresponds to a dizygotic twin pair. Data was collected during meditation period 2 (M2, as depicted in Fig. 1). **B** Mean BBI Pearson correlation between each twin participant and the remaining 7 twin participants for all 17 meditation periods. Values for co-twins are represented by red circles (∙), while values for non-twin pairs are shown as black squares ( <). Each twin group was separated during three of the following meditation periods M3, M4, M9, M10, M14, and M15. **C** Mean BBI absolute difference between each twin participant and the remaining 7 twin participants for all 17 meditation periods. Values for co-twins are represented by red circles (∙), while values for non-twin pairs are shown as black squares ( <). **D** Mean and standard error of mean BBI Pearson correlation for T2, DT2, CT2, and N2 groups (defined in Table 2) for all 17 meditation periods. Wilcoxon rank-sum test was performed (*p < 0.05). **E** Mean and standard error of mean BBI difference for T2, DT2, CT2, and N2 groups for all 17 meditation periods. Wilcoxon rank-sum test was performed (*p < 0.05).** F** Average mean BBI Pearson correlation of all pairs in T2, DT2, C2, and N2 groups for each meditation period. **G** Average mean BBI Pearson correlation for T2, DT2, C2, and N2 groups for each meditation period

Previous studies in twins have demonstrated that heart rate has a strong genetic component (Jensen et al., 2018). To explore the effect of meditation on heart rate dynamics in twins, we continuously collected BBI data in MZ and DZ twin pairs throughout the 7-day meditation retreat using a Garmin wristwatch. Shown in Fig. 8A are BBI time series from a selected meditation, M2, on Day 2 of the retreat (Fig. 1B) for twin pairs A, B, C, and D (data from twin sets E and F were unavailable, see Table 1). We observed similarities in BBI waveform shapes for both MZ (A, B, C) and DZ (D) twin pairs. The BBI baselines, however, differed for twin pair D; D maintained a baseline of ~ 1250 ms (50 BPM) while D2 was ~ 750 ms (80 BPM).

To further investigate the similarity of BBI time series for the four pairs of twins, we calculated Pearson correlation (Fig. 8B) and absolute difference (Fig. 8C) averaged over the 17 meditation periods (M1–M17). For both measures, each twin in A through D twin pairs was compared to the remaining 7 participants in the group. The red circles in each plot represent similarity indices for co-twins, while the black squares indicate values derived for non-sibling pairs. We observed a higher Pearson correlation for MZ and DZ twin pairs and a lower BBI absolute difference (signifying greater concordance) for the MZ twin pairs (Fig. 8B). However, a greater mean BBI absolute difference was observed for the DZ twin group compared to the non-pair comparisons (Fig. 8C) and can be attributed to the large difference in baseline heart rates (noted above).

Next, we compared MZ and DZ twin groups to age-matched meditating controls. We carried out analyses on 21 participants (Table 2). Ages for all participants resembled a normal distribution based on results from the Kolmogorov–Smirnov test (*D* = 0.115, *p* = 0.926) with a mean of 50.7 and a standard deviation of 14.9 years, while ages for MZ participants (*D* = 0.353, *p* = 0.358) had a mean of 48.0 and a standard deviation of 16.5 years. An independent samples *t*-test revealed no significant difference in ages between these two groups (*t* = − 0.326, *p* = 0.754), though the age of the DZ pair (76 years) was shown to significantly differ from the overall sample mean in a one-sample *t*-test (*t* = − 7.439, *p* < 0.001). The twin group included MZ twin A through C pairs (6 participants) and DZ twin pair D (2 participants). The age-matched control group included 13 participants, with one participant acting as a control for both B and D twin pairs. Figure 8D shows the mean BBI Pearson correlation for four groups of meditating pairs averaged over the 17 meditation periods. These groups include MZ twin pairs (T2), DZ twin pairs (DT2), one co-twin with an age-matched control participant (CT2), and non-twin pairs (N2).

**Table 2:** Twin and non-twin demographic data

Twin-group				Age-matched-control-group	
Pair index	Type	Gender	Age	# (male, female)	Age
A	Monozygotic twins	F	25	3 (0, 3)	34.3 ± 3.1
B		F	63	2 (0, 2)	64.0 ± 5.7
C		M	56	8 (3, 5)	49.3 ± 5.8
D	Dizygotic twins	F	76	1 (0,1)	68

The mean BBI Pearson correlations for T2, DT2, CT2, and N2 groups are 0.090, 0.060, 0.039, and 0.029, respectively. A higher mean BBI Pearson correlation was observed for T2, followed by DT2, CT2, and N2 (T2 > DT2 > CT2 > N2). A statistically significant difference was only observed between the T2 group and either co-twin/age-matched control pairs (CT2) or non-twin co-twin pairs (N2) (T2 vs. DT2, *p* = 0.086; T2 vs. C2, *p* = 0.0006, T2 vs. N2, *p* = 0.00005; DT2 vs. C2, *p* = 0.28; DT2 vs. N2, *p* = 0.081). Using the same set of groups, we determined the mean BBI absolute difference (Fig. 8E). An inverse but similar trend was observed as compared to mean BBI Pearson correlation. However, as noted previously, the DT2 group had a significantly higher mean BBI absolute difference explained by the higher baseline BBI observed for twin participant D1 (Fig. 8A). Output from mixed effects modeling confirmed that, despite adjusting for the influence of time-variation in BBI measures, the DT2 group exhibited a significantly higher BBI difference compared to the control group (*d* = 81.44, *p* = 0.043), and the T2 group exhibited a significantly lower BBI difference compared to the control group (*d* = − 101.66, *p* < 0.001), with no difference observed between the N2 group and controls. Furthermore, though the time variable itself did not exhibit a statistically significant effect, covariates testing the interaction of group identification and time showed that BBI difference significantly decreased for DT2 relative to control (*d* = − 9.20, *p* = 0.017) and significantly increased for T relative to control (*d* = 8.97, *p* = 0.002), with no significant changes in the time pattern for the N2 group relative to control.

Next, we examined the BBI Pearson correlation across the 17 meditation periods and found an increasing trend from − 0.0067 in M1 to 0.0945 in M17 for all groups (Fig. 8E). A separate analysis revealed that the MZ twin group (T2) significantly exceeded all other groups in Pearson correlation, reaching a value of 0.299 in M17 (Fig. 8G). This finding corroborates previous studies indicating a strong genetic influence on heart rate dynamics (Jensen et al., 2018).

### Neurological Correlates in Twin Pairs During a 7-Day Intensive Meditation Retreat

**Fig. 9:**
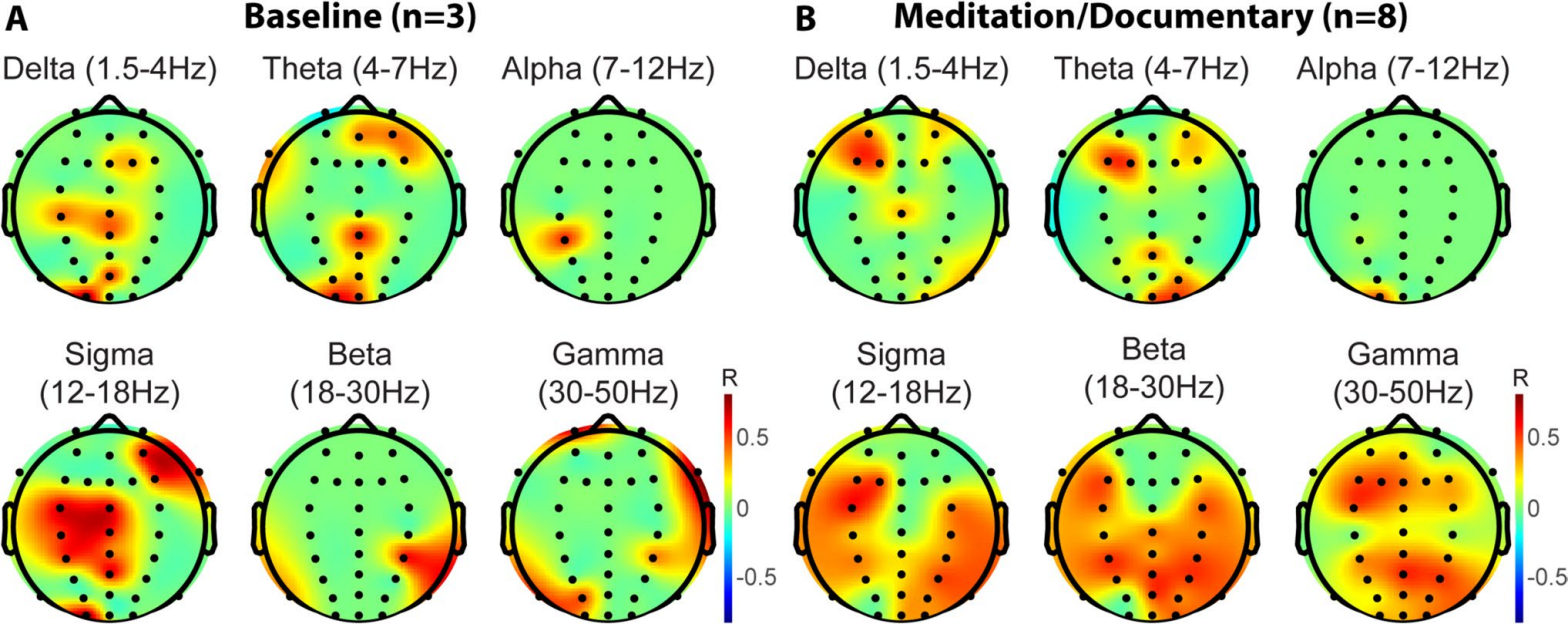
Scalp maps of correlation *R*-values significantly (*p* = 0.05) higher than randomly paired twins by bootstrap statistics. **A** Spectral correlations between twins during the joint baseline/T0 meditation before the retreat. **B** Spectral correlations during a retreat meditation while co-twins were separated and one was listening to a documentary

Figure 9 shows mean correlation *R*-values across MZ twin pairs (*n* = 3 for baseline/T0, *n* = 8 for meditation/documentary) that were significant by permutation of the sham pairings (*p* < 0.05). Non-green voxels in the scalp maps represent mean correlation values from twin pairs greater than 95% of all sham pair averages (*p* = 0.05). For the baseline/T0 condition in which co-twins meditated together in the same room, a correlation was observed in the sigma (12–18 Hz) range on the left hemisphere as well as in the right frontal theta (4–7 Hz), sigma and gamma (30–50 Hz), left occipital delta (1.5–4 Hz), theta, sigma and gamma, and right posterior beta (18–30 Hz) (Fig. 9A). During the meditation/documentary condition, when only one twin was meditating, and their co-twin was listening to a documentary in a different room, more areas of the brain were significantly correlated compared to sham pairings, including correlations in sigma, beta, and gamma across most of the head. At the same time, delta and theta were confined mostly to the left frontal areas (Fig. 9B). Interestingly, the alpha band did not correlate significantly in either condition. These data indicate that twins share features of ongoing EEG activity during rest and while engaged in different activities. However, we found that, especially in the higher cognitive frequency ranges of beta and gamma, more activity was shared while one twin meditated, suggesting a highly transmissible mental state.

## Discussion

Meditation and other mind–body practices have been shown to improve the stress response and enhance both health and resilience. These effects are now being actively investigated in a retreat-style setting (Alvarez-Lopez et al., 2022; Chandran et al., 2021; Epel et al., 2016; Zuniga-Hertz et al., 2023). Twin studies offer a powerful method for exploring the interplay between nature and nurture. To date, the effect of meditation on twins has been largely unexplored. Here, we examined molecular, physiological, and neurological correlates in a small cohort of meditating twins. To our knowledge, this study represents the first of its kind. Throughout the 7-day meditation retreat, an examination of gene alterations revealed that the most significant shift in the group occurred at the beginning of the retreat, with a gradual decrease throughout the week. Examination of cytokines and other selective plasma proteins revealed changes in both pro- and anti-inflammatory factors. Metabolomic studies demonstrated that twin pairs clustered together at each time point. Physiological studies confirmed previous findings showing high heritability of heart rate (Jensen et al., 2018). Finally, brain mapping data suggest a greater neurophysiological correlation among twin pairs than unrelated pairs. In summary, these data provide a first glimpse at the overall effects of meditation in a small twin cohort. Future studies, including a larger number of MZ and DZ twin sets, will allow us to expand on this work and further investigate the biological, physiological, and neurological connectedness in individuals with a shared genetic background.

Gene transcription studies demonstrated a clustering of genes by twin set (Fig. 2A) that can be explained by similar genotypic characteristics of twin pairs. By time, the largest variation of changed genes in the group occurred at T1 on Day 3 of the retreat (Fig. 2B), approximately at the halfway point (Fig. 1). Interestingly, this variation decreased by T3 on Day 7 of the week-long retreat (Fig. 2B). Based on these observations, we hypothesize that the retreat environment resulted in an immediate increase in the stress response, with a renormalization in phenotype over the 7 days. GO enrichment demonstrated an increase in multiple mitochondrial-specific processes, including oxidative phosphorylation, electron transport chain, cellular respiration, and ATP synthesis in time points T1 and T2 relative to baseline (Fig. 4A, B). Consistent with these findings, a previous study by Bhasin et al. (2013) showed enhanced expression of several genes, including those linked to energy metabolism and mitochondrial function, following a relaxation response-eliciting practice. Additionally, meditation has been found to induce changes in brain activity, including deactivating some areas and activating others (Bauer et al., 2019). Thus, we speculate that these changes result in a shift in fuel sources used by the brain and the body, with the brain and body using energy in unique ways during meditative practice. It is important to note that while these trends are indeed interesting, we did observe some variability of basal gene expression between twin pairs across all time-dependent groups (Fig. 2A), thus limiting the accuracy of DEGs detection. One major limitation of this study is the small number of twin pairs. Enrolling a larger number of twin participants to increase the group size could improve the statistical power of differential expression estimates and downstream predictive analyses and would strengthen the conclusions. A previous study carried out in long-term and short-term practitioners of the relaxation response demonstrated enrichment of mitochondrial-specific activities, including cellular metabolism, oxidative phosphorylation, and generation of reactive oxygen species (Dusek et al., 2008). A more recent study by Bhasin et al. (2013) found an upregulation in ATP activity and a downregulation of apoptotic-regulating genes in practitioners of the relaxation response. It is worth mentioning that the meditation techniques used in the present study are more like Kundalini- and Vipassana-type practices than relaxation practices. The Kundalini experience is often described as a surge of spiritual energy that brings about heightened awareness, inner peace, and a deep connection with one’s true self. Vipassana meditation, originating from Buddhist traditions, is a practice of both mindfulness and insight in which one observes the sensations of the body with heightened awareness, fostering a deep understanding of the impermanence and interconnected nature of all experiences (Chattopadhyay, 2021). Despite the differences between these two techniques and relaxation practices, there appears to be some overlap in gene expression changes that occur.

Due to the multidimensional nature of meditation interventions, a broad approach was taken to explore the immunological, hormonal, and enzymatic milieu of participant plasma in relation to the meditation intervention. Surprisingly, despite the genetic similarity in twin pairs, variation in the human immune system is mostly driven by non-heritable influences (e.g., viral infection, inflammatory triggers) (Brodin et al., 2015). While we observed variation in the expression of plasma cytokines between twin pairs, trends were seen in both pro- and anti-inflammatory pathways (Fig. 5). The largest increase was observed in MMP-9, an enzyme involved in tissue remodeling and inflammation. Interestingly, it has been suggested that MMP-9 may play an important role in the restructuring of synaptic networks in the adult brain (Reinhard et al., 2015). MMP-9 regulates the degradation of extracellular matrix (ECM) in a broad spectrum of beneficial physiological processes that involve tissue remodeling, including those that occur during embryonic and neonatal development (Yabluchanskiy et al., 2013). In addition, this protein is involved in modulating immune function (McMillan et al., 2004). It is noteworthy that while MMP-9 has several important physiological functions, it can, under some circumstances, contribute to pathological conditions such as autoimmune disease and cancer (Cabral-Pacheco et al., 2020). Of the five out of 105 surveyed proteins that decreased from baseline/T0 to T3, adiponectin displayed the largest relative decrease. Adiponectin, an adipokine produced by adipose tissue, significantly regulates glucose levels and fatty acid breakdown (Yanai & Yoshida, 2019). Adiponectin has also been shown to have anti-inflammatory properties (Nguyen, 2020). More than half (7) of the 12 twin participants demonstrated decreases in adiponectin, suggesting an increase in inflammation. Interestingly, individual C2 demonstrated widespread decreases in most cytokines, except IL-18BP, a factor important in regulating plasma levels of IL-18, a pro-inflammatory cytokine. This pattern was not observed in their co-twin. SERPINE1, an immune-related protein shown to have roles in both cellular senescence (Khan et al., 2017) and cancer (Li et al., 2018), decreased following the 7-day retreat, suggesting that the retreat experience may have anti-aging and anti-cancer effects. Finally, OPN, a multi-faceted pro-inflammatory chemokine (Lund et al., 2009), and PGDF-AA, a protein with a role in wound healing and repair, were also reduced in post-meditation plasma. These data indicate trends for both an increase and a decrease in inflammation after the 7-day retreat. Given the critical role of inflammatory signaling in growth and regeneration (Cooke, 2019), these trends are consistent with tissue remodeling and repair. Interestingly, a decrease in apoptotic processes was observed in GO analyses (Fig. 4). Thus, it is tempting to speculate that a negative feedback loop occurred, such that an increase in inflammation and tissue remodeling gene products results in a suppression of the corresponding genes. While these data are intriguing, given the small number of participants, statistical significance could not be established. Future studies involving a larger cohort will be needed to confirm these findings.

Untargeted metabolomics analysis across time points and twin pairs revealed differential regulation of several families of metabolites (Fig. 6). Among the highest impact on the PLS-DA model were several plasmalogens, a class of phospholipids. Six plasmalogens increased as a result of the retreat, including (1-(1-enyl-steroyl)−2-oleoyl-GPE, (1-(1-enyl-steroyl)−2-linoleoyl-GPE, (1-(1-enyl-steroyl)−2-arachidonoyl-GPE, (1-(1-enyl-palmitoyl)−2-oleoyl-GPE, (1-(1-enyl-palmitoyl)−2-arachidonoyl-GPE, and (1-(1-enyl-palmitoyl)−2-linoleoyl-GPE. Plasmalogen supplementation has been associated with the modulation of cognitive performance and can decrease neuroinflammation (Decandia et al., 2023).

The breakdown of fatty acids for energy production is a key component of overall energy metabolism. Both TCDCA (through fat digestion and absorption) and acyl-carnitines (through fatty acid transport and oxidation) use fats as an energy source. The present data suggest that changes in metabolic efficiency may occur after an intensive mediation experience. TCDCA, which has been reported as an anti-inflammatory-related metabolite (Xu et al., 2023), was significantly decreased from baseline/T0 to T3, indicating dysregulation of fat usage. Another metabolite of interest that is involved in the propanoyl-coenyzme A (CoA) metabolic pathway, BAIBA, plays a role in valine, leucine, and isoleucine degradation; is typically produced by the catabolism of skeletal muscle; and increases during exercise. We observed a trending decrease in BAIBA from baseline/T0 to T3 (Fig. 6). In addition, decreases in constituents of acyl-carnitine metabolism were also observed (Fig. 7). Such decreases indicate a decrease in fatty acid regulation and cellular energy. While exercise is known to induce acyl-carnitine metabolism (Hiatt et al., 1989), the meditation experience had the opposite effect. Thus, despite this form of meditation practice acting as a potential stressor with the induction of selected inflammatory pathways, it does not exert these effects using the same mechanisms as exercise. Interestingly, an increase in several acyl-carnitines was associated with replicative senescence in human myoblasts (Baraibar et al., 2016), suggesting that the decrease observed here may be consistent with an “anti-aging” effect.

Other broad changes were observed in larger metabolite groups, such as increases in PC metabolism, gamma-glutamyl cycle, and tryptophan metabolism following the meditation intervention. Tryptophan metabolism is a key component of the gut-brain axis, shown to mediate communication between the periphery and the central nervous system (Gao et al., 2020). As an essential amino acid, tryptophan can modulate neuroendocrine and intestinal immune responses and is a precursor of serotonin and melatonin. Previous studies have demonstrated that long-term meditators have higher serum levels of melatonin (Tooley et al., 2000) and its precursor serotonin (Thambyrajah et al., 2023) compared to controls. The vagus nerve is a tangential key signaling pathway between the central and peripheral nervous systems. Increases in vagal tone have been associated with positive emotions and promoting positive feelings. Activation of the vagus nerve by vagal nerve stimulation results in increased serotonin production (Ben-Menachem et al., 1995) and meditation is known to stimulate the vagus nerve by increasing vagal tone (Paccione et al., 2022). PC has been shown to have anti-inflammatory properties (Eros et al., 2009; Treede et al., 2007). Thus, an increase in PC following meditation may suggest that a decrease in stress may also reduce inflammation (Treede et al., 2007). Lastly, an increase in the gamma-glutamyl cycle indicates increased glutathione production, a powerful anti-oxidation pathway, and may act as a proxy for alterations in mitochondrial energy regulation and the generation of reactive oxygen species. Lowering stress through meditation can potentially impact antioxidant generation and downstream bodily responses to oxidative stress.

In previous work, the heritability of heart rate has been well-documented in MZ twins (Jensen et al., 2018). Our study extends these findings by demonstrating a highly correlated BBI time series among meditating MZ twins (Fig. 8A). However, we observed a notable divergence in BBI baseline readings in the single DZ twin pair in our sample. This divergence was significant when compared to MZ twins and to control and non-twin groups across all 17 meditation sessions (Fig. 8C, E). The limited representation of DZ twins in this study precludes any definitive conclusions regarding these differences, and future investigations would benefit from a balanced number of MZ and DZ twin pairs for a more robust comparative analysis.

Intriguingly, our DZ twin group exhibited a higher mean BBI Pearson correlation than observed in both control and non-twin groups (Fig. 8D) and significantly greater improvements in BBI coherence across meditation periods. Although DZ twins are not genetically identical, they share certain genetic traits and environmental exposures in addition to their lifelong relationships. This point raises the question of whether heart rate (HR) dynamics may be influenced or even enhanced in pairs who have established long-term social bonds, such as spouses or close friends. The methodologies developed in this study could serve as tools for exploring such questions further.

Unexpectedly, we observed an increased correlation in HR dynamics among pairs over the duration of the week-long meditation retreat; this increase was most pronounced in the MZ twins. This finding suggests that while HR correlations can be cultivated, the process may be more readily facilitated in genetically identical individuals.

Our methodology leveraged wearable technology, specifically Garmin devices utilizing photoplethysmography (PPG), to assess heart rate. Unlike traditional electrocardiography (ECG), which relies on skin electrodes to measure cardiac electrical activity, PPG gauges changes in blood volume via light absorption or reflection through the skin. Although both technologies can effectively measure heart rate, they may yield different results due to factors such as measurement sites, signal processing, motion artifacts, and physiological parameters. Nevertheless, our study design focused on HR dynamics of larger time scales (correlations at 60-s epochs), minimizing the need for millisecond accuracy, and on meditation sessions where participants remained largely static, attenuating any potential motion artifacts and thus enhancing the reliability of our PPG-derived readings. To substantiate the PPG data, we cross-referenced it with ECG measurements from a parallel study, revealing comparable BBI readings between FirstBeat Bodyguard 2 and Garmin VivoSmart 4 devices (data not shown). This concordance strengthens the validity of our findings and supports the feasibility of large-scale, low-cost studies on heart rate dynamics using wearable technology.

Quantitative electroencephalography (qEEG) has been used to outline the neurophysiological correlates of meditation. These studies have primarily focused on oscillatory patterns induced by different styles of meditation (reviewed in Fell et al. (2010)). A common pattern observed for several different forms of meditation is a slowing of alpha rhythms (8–12 Hz), an increase in alpha power, and a general increase in theta activity (3–8 Hz). A separate set of studies has shown that EEG oscillatory patterns are highly variable depending on the meditation practice (Travis & Shear, 2010).

Previous studies in twins have shown brain activity to be a highly heritable trait (van Beijsterveldt & van Baal, 2002) and stable over time (Pollock et al., 1991; Salinsky et al., 1991). While both brain activity and function have been explored during numerous meditative practices, data is lacking in meditating twins. To this end, we measured brain activity in twins meditating together and in twins separated and simultaneously either meditating or listening to a documentary. Simultaneous EEG studies have generally focused on shared attention or social interactions (Liu et al., 2018). Our baseline condition is a type of shared-attention paradigm, while all other paradigms have relied on visual rather than auditory attention. Previous studies have demonstrated that joint interactive attention was associated with a decreased high alpha power (Lachat et al., 2012), and face-to-face student interactions resulted in greater alpha coherence that also correlated with class engagement (Dikker et al., 2017). Our EEG data shown in Fig. 9 demonstrate that MZ twins have more similar dynamics in spectral power during a shared meditation experience than randomly paired twins. Interestingly, alpha was the one frequency band that did not show any significant correlation between twins, even though it has been implicated in both meditation and shared-attention studies. It is possible that splitting up the alpha band would have affected upper alpha frequencies. Another possibility is that the meditation paradigm, being non-social and passive, induced correlations in different frequency bands. Surprisingly, we see that coupling was even more widespread when only one twin was meditating, and their co-twin was in a different room listening to a documentary. This condition is not, to our knowledge, like any published studies and stretches our limits of possibility to imagine how they could be more neurally connected while in separate rooms and doing different things. Perhaps the retreat itself caused twin pairs to become more connected than they were at the beginning of the retreat. The number of twin pairs was also extremely small for both groups and particularly for the baseline group. Thus, any interpretations of the present results would need to be confirmed with future studies employing time-locked EEG recordings, a larger number of twin pairs, and the addition of non-related pairs analyzed under the same experimental conditions.

### Limitations and Future Research

This pilot study represents a comprehensive first attempt at investigating the effect of intensive meditation in a retreat setting in a twin cohort. While the data show some interesting physiological, neurological, and biological trends, several limitations must be noted. Regarding the retreat setting and due to logistical reasons, blood samples were collected after a short fast (30 min to 1.5 h), not an overnight fast, potentially leading to some variability in blood samples. In addition, due to variations in the daily meditation schedule, blood samples were not collected at the exact time of day for all twin participants. Thus, some variability in blood samples across time points may have been introduced due to circadian rhythm effects. Gene expression studies showed that a small number of twins drove large differences in a few selected genes and may not reflect the whole group. Also, due to the small number of twin sets, extensive statistical analysis could not be carried out for the biological and EEG studies. Additionally, only one DZ twin set was included in this work, and their age was significantly greater than that of the other participants. While the effect of sex could not be assessed in the present study with only one male twin set, a previous study from our group demonstrated that there was no effect of the dynamics of meditation on sex (Zuniga-Hertz et al., 2023). Similarly, while age could not be assessed in this small twin cohort, a minimal effect on age was observed in a previous study (Zuniga-Hertz et al., 2023). That being said, future studies will explore both age and sex effects in meditating twins.

The present study findings reveal changes in pro- and anti-inflammatory blood factors at the transcriptomic and protein levels in a small cohort of fully or partially meditating twins during an intensive 7-day meditation retreat. Metabolomic studies in the group are consistent with these findings. Heart rate dynamics and qEEG studies both confirm the high heritability of heart rate and brain activity and raise the question of whether both may be influenced in pairs with long-term bonds. Future studies, including a greater number of twin participants, equal numbers of MZ and DZ twin pairs, and both pre- and post-retreat data collection, are needed to confirm these findings.

In summary, this pilot study (In, 2017) provides a glimpse of the molecular, physiological, and neurophysiological features of a meditating twin cohort. While recruitment methods were adequate, in the follow-up study and in addition to recruiting a larger number of MZ and DZ twin sets, we will exclude one co-twin from each pair from participation in the retreat. These changes will allow for robust statistical analysis of molecular outcomes and more clearly defined effects of meditation retreat interventions.

## Supplementary Information

Below is the link to the electronic supplementary material.

Supplementary file1 (PDF 39 KB)

Supplementary file2 (PDF 32 KB)

Supplementary file3 (XLSX 11977 KB)

Supplementary file4 (XLSX 26 KB)

Supplementary file5 (XLSX 15 KB)

Supplementary file6 (XLSX 34 KB)

Supplementary file7 (XLSX 34 KB)

## Data Availability

RNA-seq data are available in the NCBI Gene Expression Omnibus (GEO) repository (accession number GSE252632). DEGs are included in Supplementary Table S3.

## Declarations

### Use of Artificial Intelligence

AI was not used.
