# Supplementary material for: Multidimensional Analysis of Twin Sets During an Intensive Week-Long Meditation Retreat: A Pilot Study
Source: Mindfulness (N Y). 2025 May 7;16(6):1634–55. doi: 10.1007/s12671-025-02584-x (PMC12170707; doi:10.1007/s12671-025-02584-x)
Supplement: Supplementary file 1 — Supplementary file1 (PDF 39 KB) [file 12671_2025_2584_MOESM1_ESM.pdf]

Supplementary Table S1. Bioinformatics resources.

Description	Source	Identifier	URL
**Trimmomatic**	[1]	Version 0.39	http://www.usadellab.org/cms/?page=trimmomatic
**STAR**	[2]	Version 2.7.10b	https://github.com/alexdobin/STAR
**Tximeta**	[3]	Version 3.11	https://www.bioconductor.org/packages/release/bioc/html/tximeta.html
**FastQC**	Babraham Bioinformatics	Version 0.12.0	https://www.bioinformatics.babraham.ac.uk/projects/fastqc/
**MultiQC**	Stockholm University, Stockholm, Sweden [4]	Version 1.9	https://multiqc.info/docs/
**PCAtools**	Blighe K, Lun A	Version 3.18	https://bioconductor.org/packages/PCAtools
**DESeq2**	[5]	Version 1.36.0	https://www.bioconductor.org/packages/devel/bioc/vignettes/DESeq2/inst/doc/DESeq2.html
**Human genome**	Gencode	Release 43 (GRCh38.p13)	https://www.gencodegenes.org/human
**clusterProfiler Bioconductor package**	[6]	Release 4.8.1	https://bioconductor.org/packages/release/bioc/html/clusterProfiler.html
**Kyoto Encyclopedia of Genes and Genomes (KEGG)**	[7]	Release 106.0	https://www.kegg.jp/
**Gene Ontology (GO)**	[8]	release 2023-05-10	http://geneontology.org/
**R programming language**		Version 4.3.1	https://www.r-project.org/
**Bioconductor**	[9]	Version 3.17	https://bioconductor.org/

1. Bolger, A.M., M. Lohse, and B. Usadel, *Trimmomatic: a flexible trimmer for Illumina sequence data.* Bioinformatics, 2014. **30**(15): p. 2114-20.

2. Dobin, A., et al., *STAR: ultrafast universal RNA-seq aligner.* Bioinformatics, 2013. **29**(1): p. 15-21.

3. Love, M.I., et al., *Tximeta: Reference sequence checksums for provenance identification in RNA-seq.* PLoS Comput Biol, 2020. **16**(2): p. e1007664.

4. Ewels, P., et al., *MultiQC: summarize analysis results for multiple tools and samples in a single report.* Bioinformatics, 2016. **32**(19): p. 3047-8.

5. Love, M.I., W. Huber, and S. Anders, *Moderated estimation of fold change and dispersion for RNA-seq data with DESeq2.* Genome Biol, 2014. **15**(12): p. 550.

6. Wu, T., et al., *clusterProfiler 4.0: A universal enrichment tool for interpreting omics data.* The Innovation, 2021. **2**(3): p. 100141.

7. Kanehisa, M., et al., *KEGG for taxonomy-based analysis of pathways and genomes.* Nucleic Acids Research, 2023. **51**(D1): p. D587-D592.

8. Ashburner, M., et al., *Gene ontology: tool for the unification of biology. The Gene Ontology Consortium.* Nat Genet, 2000. **25**(1): p. 25-9.

9. Gentleman, R.C., et al., *Bioconductor: open software development for computational biology and bioinformatics.* Genome Biol, 2004. **5**(10): p. R80.
