# Supplementary material for: Multidimensional Analysis of Twin Sets During an Intensive Week-Long Meditation Retreat: A Pilot Study
Source: Mindfulness (N Y). 2025 May 7;16(6):1634–55. doi: 10.1007/s12671-025-02584-x (PMC12170707; doi:10.1007/s12671-025-02584-x)
Supplement: Supplementary file 2 — Supplementary file2 (PDF 32 KB) [file 12671_2025_2584_MOESM2_ESM.pdf]

Supplementary Table S2. Experimental design for Twin and Control Pairwise Comparison

	Label	Experimental Condition	Number of Pairs
Twin Group	T2	Monozygotic (MZ) twins; both meditating	28
	T1	MZ twins; one meditating, one watching documentary	7
	DT2	Dizygotic (DZ) twins; both meditating	11
	DT1	DZ twins; one meditating, one watching documentary	3
	N2	Two participants from different twin pairs; both meditating	237
	N1	Two participants from different twin pairs; one meditating, one watching documentary	44
	MT	Missing data in twin group	146
Control Group	CT2	One twin group participant vs. age-matched control; both meditating	246
	C1	One twin group participant vs. age-matched control; one meditating, one watching documentary	25
	MC	Missing data in control group	205
